# Divergence in isoprenoid emissions among subtropical broadleaf trees: linking photosynthesis, leaf economics, and volatile defense for forest management

**DOI:** 10.48130/forres-0026-0023

**Published:** 2026-07-29

**Authors:** Yimiao Mao, Hang Yao, Yali Yuan, Chao Yu, Meiling Wang, Fei Ding, Guomo Zhou, Zhihong Sun

**Affiliations:** 1 Institute of Agriculture and Biology, Liaocheng University, Liaocheng, Shandong 251000, China; 2 College of Horticulture Science, Zhejiang A&F University, Hangzhou, Zhejiang 311300, China; 3 Zhejiang Academy of Agricultural Sciences, Hangzhou, Zhejiang 310021, China; 4 School of Environmental and Resources Science, Zhejiang A&F University, Hangzhou, Zhejiang 311300, China

**Keywords:** BVOCs, Leaf economic spectrum, Photosynthetic electron transport, Adaptive strategy, Subtropical forestry, Forest management, Tree physiology

## Abstract

Biogenic volatile organic compound (BVOC) emissions are pivotal in plant–environment interactions, yet their intrinsic linkages with photosynthesis and leaf economics across diverse tree species remain poorly integrated. This study investigated seven subtropical broadleaf species spanning distinct lineages to unravel how BVOC chemotypes (isoprene- vs. monoterpene-dominance) align with photosynthetic energy use and leaf structural traits. Using controlled measurements on excised branches to isolate physiological relationships, we combined TD-GC-MS, gas-exchange, and leaf trait analyses. The results revealed strong divergence in BVOC chemotypes that was broadly coherent with species phylogeny and ecological strategy. Isoprene-dominant (ISO) species exhibited higher photosynthetic rates (*A*), greater electron transport capacity (*J*_max_), and elevated *J*_max_/*V*_cmax_ ratios, consistent with the hypothesis that isoprene synthesis functions as a photosynthetic overflow mechanism for energy dissipation. In contrast, monoterpene-dominant (MTS) species displayed higher specific leaf weight (SLW) and relied on stored and induced emissions, reflecting a conservative, defense-oriented strategy. BVOC profiles were strongly correlated with SLW and photosynthetic capacity, highlighting a coordinated 'structure–function–volatile' adaptive syndrome. These findings suggest that BVOC emissions may be embedded within leaf economic strategies and photosynthetic networks, providing a basis to help refine next-generation BVOC models by integrating leaf structural traits (e.g., SLW) and photosynthetic electron transport capacity (*J*_max_) into model parameterization.

## Introduction

Plant-derived biogenic volatile organic compounds (BVOCs) are a diverse group of low-molecular-weight organic compounds emitted during plant growth and development. The major classes of BVOCs, categorized according to their biosynthesis pathways, include terpenoids (such as isoprene, monoterpenes, and sesquiterpenes), compounds derived from fatty acid metabolism (so-called green leaf volatiles [GLVs]), and aromatic compounds^[1−3]^. Terrestrial ecosystems emit about 1 billion tons of carbon equivalent in BVOCs annually, accounting for over 90% of global VOC emissions^[4]^. Among these, isoprenoids, particularly isoprene and monoterpenes, constitute more than 75% of the total BVOC emissions^[5,6]^. These compounds play key roles in ecological processes and influence ecosystem structure and function through biogeochemical feedbacks^[6−9]^.

Throughout their life cycle, plants face a multitude of biotic and abiotic stresses, including herbivory, pathogens, drought, extreme temperatures, and salinity. BVOCs serve key functions in mitigating these stresses and facilitating reproduction^[6,10]^. In response to biotic stress, BVOCs operate through direct and indirect defenses. Plants can directly emit terpenoids and GLVs to effectively deter pests and pathogens. For instance, monoterpenes such as limonene and eucalyptol exhibit insecticidal and repellent properties by inhibiting insect and microbial activity^[5,11,12]^. Similarly, GLVs such as *cis*-3-hexenol exhibit strong antimicrobial effects against bacteria and fungi, while *trans*-2-hexenal repels insect larvae to reduce herbivory^[13]^. Some phenylpropanoids, including eugenol, also exhibit broad-spectrum antimicrobial activity, inhibiting mycelial growth^[14]^. For indirect defense, plants release specific BVOCs for chemical signaling. For example, maize attacked by *Ostrinia furnacalis* emits *cis*-3-hexenyl acetate and terpenoids to attract parasitic wasps (*Trichogramma*)^[15]^. Wounded *Arabidopsis thaliana* releases *cis*-3-hexenol, which primes neighboring plants via jasmonic acid and ethylene pathways, inducing early defense gene expression and forming a 'group immune' response^[16]^. BVOCs also enhance reproduction by attracting pollinators such as bees and butterflies^[17,18]^.

Under abiotic stress, BVOCs play critical protective roles in plants. For instance, many tree species (e.g., poplar, oak) emit substantial amounts of isoprene in response to high temperature or intense light^[2,19]^. Beyond its well-established function in stabilizing chloroplast membranes, emerging evidence suggests that isoprene may also act as a hormone-like signal, modulating cellular metabolism and stress-response pathways to enhance the resilience of photosynthetic tissues^[20−22]^. Meanwhile, certain monoterpenes function as effective lipophilic antioxidants and can act as defense primers by inducing the expression of stress-related genes, with some even facilitating interplant communication^[21,23]^. Additionally, sesquiterpenes and aromatic compounds contribute to drought tolerance by modulating osmotic potential, stabilizing membranes, and reducing evaporative water loss^[24,25]^.

BVOC biosynthesis is an integral part of plant secondary metabolism. Terpenoid synthesis occurs mainly via two pathways: the mevalonic acid (MVA) pathway in the cytoplasm and the methylerythritol phosphate (MEP) pathway in plastids, both producing the universal five-carbon precursors, isopentenyl diphosphate (IPP) and dimethylallyl diphosphate (DMAPP)^[1,26]^. The MVA pathway predominantly synthesizes sesquiterpenes, triterpenes, sterols, and others, whereas the MEP pathway yields isoprene, monoterpenes, diterpenes, and carotenoids^[2,27]^. Aromatic compounds are derived from phenylalanine via phenylalanine ammonia-lyase (PAL), leading to volatiles such as benzaldehyde, phenethyl alcohol, and methyl salicylate^[28]^. GLVs are generated through the lipoxygenase (LOX) pathway, which oxidizes and cleaves fatty acids into C6–C9 aldehydes, alcohols, and esters, often released rapidly upon damage^[29,30]^.

Rather than representing mere carbon loss, BVOCs reflect a metabolic investment by plants. Their synthesis and emission are tightly coupled to photosynthetic carbon fixation, which supplies necessary substrates and energy (ATP, NADPH)^[31]^. Thus, BVOC emission constitutes a consumptive allocation of photosynthetically fixed carbon, revealing strategic trade-offs in resource use^[31,32]^. Although all three major classes of BVOCs are widespread, emission profiles (composition, rates, and inducibility) are highly species-specific and phylogenetically conserved^[7,33]^. For example, Pinaceae species are high monoterpene emitters (e.g., *α*-pinene); Salicaceae (*Populus*), Fabaceae, Moraceae (*Ficus*), and Fagaceae (*Quercus*) typically emit high levels of isoprene; and Magnoliaceae and Lauraceae chiefly release monoterpenes^[33,34]^. These traits ultimately underscore the tight coupling between BVOC emission and photosynthetic carbon fixation^[2]^.

Environmental selection can drive convergent evolution in BVOC emissions across disparate lineages. For instance, in Mediterranean ecosystems characterized by drought and high irradiance, distantly related families (e.g., Oleaceae*,* Myrtaceae) have independently evolved high-light-induced isoprene emission, leveraging its thermotolerance properties^[2,35]^. Similar adaptive elevations in isoprenoid emissions are observed in lineages inhabiting hot, arid savannas^[33,36]^. Conversely, at the interspecific level, BVOC emission patterns are often phylogenetically conserved and reflect long-term ecological adaptation^[37]^. However, the physiological mechanisms linking these macro-evolutionary patterns to underlying photosynthetic performance and leaf economic strategies remain fragmented. Specifically, how the coordinated interplay of photosynthetic energy allocation, leaf structural investment, and BVOC chemistry coalesces into distinct adaptive syndromes remains to be fully elucidated^[18,38]^.

To bridge this gap, we selected seven subtropical broadleaf woody species spanning diverse families and ecological habits. By integrating measurements of BVOC emission profiles, photosynthetic gas-exchange parameters, and key leaf structural traits under controlled conditions, this study aimed to: (1) uncover the intrinsic linkages between BVOC chemotypes (isoprene- vs. monoterpene-dominance) and core physiological traits; and (2) synthesize these relationships into a coherent 'structure–function–volatile' framework explaining divergent adaptive strategies in forest trees. These findings provide a mechanistic basis for incorporating plant functional traits into predictions of forest-atmosphere interactions under environmental change.

## Materials and methods

### Species selection and study design

This study employed a mechanism-focused comparative approach to elucidate the intrinsic relationships between BVOC emission profiles and core plant functional traits. We selected seven common, co-occurring subtropical woody species from diverse families (*Machilus leptophylla*, *Carya illinoinensis*, *Choerospondias axillaris*, *Liriodendron chinense*, *Edgeworthia chrysantha*, *Wisteria sinensis*, and *Ormosia hosiei*) to capture broad variation in phylogeny, growth form, and leaf economic strategy (Table 1). Critically, the chosen species are characterized as low-to-moderate BVOC emitters, based on the emission rate classifications reported in the MEGAN2.1 model framework^[4]^ and supported by empirical data from diverse regional studies^[39−41]^. By focusing on this range, we aimed to reveal fundamental trait-emission linkages that might be obscured by the disproportionately high metabolic flux of hyper-emitters (e.g., some *Populus* or *Quercus* species), thereby addressing a knowledge gap for non-extreme emitters.

**Table 1:** Leaf functional traits and ecological characteristics of seven woody species.

Species	Family	Genus	Leaf water content (LWC, %)	Specific leaf dry weight (SLW, g·m^−2^)	Specific leaf area (SLA, cm^2^·g^−1^)	Ecological niche
*Machilus leptophylla*	Lauraceae	*Machilus*	59.945 ± 0.743e	95.138 ± 3.69a	105.883 ± 3.993c	ST, CT; evergreen understory tree in subtropical forests; prefers moist, well-drained soils
*Carya* *illinoinensis*	Juglandaceae	*Carya*	59.331 ± 0.623e	67.961 ± 4.007bc	149.701 ± 8.718b	CT; introduced deciduous tree, cultivated, adapted to full sun and deep soils
*Choerospondias* *axillaris*	Anacardiaceae	*Choerospondias*	59.1 ± 1.043e	73.674 ± 4.081b	137.784 ± 7.432bc	LT, ST; light-demanding and fast-growing deciduous tree, common on forest margins and open slopes
*Liriodendron* *chinense*	Magnoliaceae	*Liriodendron*	71.424 ± 1.076b	57.852 ± 4.23c	177.605 ± 13.222b	LT, HT, CT; fast-growing, pioneer deciduous tree of valleys and moist mountainous forests
*Edgeworthia* *chrysantha*	Thymelaeaceae	*Edgeworthia*	81.657 ± 0.346a	39.692 ± 2.254d	255.901 ± 14.084a	LT, ST; deciduous understory shrub, tolerates semi-shade; often found along streams or in moist ravines
*Wisteria* *sinensis*	Fabaceae	*Wisteria*	67.593 ± 0.975c	36.846 ± 3.01d	281.382 ± 24.679a	LT, ST, CT, DT, WT; light-demanding deciduous liana, climbing on forest edges, open areas, and riparian zones
*Ormosia* *hosiei*	Fabaceae	*Ormosia*	63.953 ± 1.604d	70.813 ± 5.667b	145.939 ± 11.994bc	LT, ST, CT; semi-evergreen tree of mixed broadleaf forests, tolerating partial shade
LT, light-tolerant; ST, shade-tolerant; HT, heat-tolerant; CT, cold-tolerant; DT, drought-tolerant; WT, waterlogging-tolerant. Values are mean ± SE (*n* = 6). Different lowercase letters with a column indicate significant differences (Tukey's test, *p* < 0.05).						

### Plant material sampling and acclimation

All plant materials were obtained from the Donghu Campus of Zhejiang A&F University (119°43' E, 30°15' N). Sampling was conducted in July 2023 (peak growing season) from three healthy, mature individuals per species. From each individual, two sun-exposed branches were excised, yielding six replicates per species (*n* = 6). This hierarchical design, with individual trees included as a random factor in all statistical analyses, follows established protocols for comparative BVOC and leaf physiology studies^[42,43]^.

To standardize conditions for rigorous physiological comparison, we used excised branches under controlled laboratory settings^[42,43]^. Immediately after excision, healthy sun-exposed branches (> 60 cm) were recut underwater to 10–15 cm to prevent xylem embolism and transported with stems submerged. This procedure maintains xylem integrity and preserves key physiological functions, including photosynthetic rates, stomatal conductance, and isoprene emissions, at levels comparable to intact attached branches^[44−47]^. A 30-min acclimation period was selected based on preliminary tests showing that this duration allowed stomatal stabilization after excision while minimizing detachment stress, consistent with established excised-branch protocols^[42,43]^.

For species with pressurized storage structures, excision may transiently elevate emissions. We controlled this potential bias through uniform handling across species, randomizing any artifacts and mixed-effects models with individual trees as a random factor, ensuring valid comparative inference.

### Volatile collection and analysis

#### BVOC sampling under controlled conditions

All BVOC sampling was conducted indoors under strictly controlled and monitored environmental conditions: temperature (28 °C), relative humidity (65%−75%), CO_2_ concentration (ambient, ~420 μmol·mol^−1^), and a saturating photosynthetic photon flux density (PPFD of 1,050 μmol·m^−2^·s^−1^) provided by a calibrated LED plant growth light (model C21GL-AE26-15W-A, SanSi, China).

A custom-made semi-open transparent glass chamber (diameter 10.5 cm, length 15 cm, 95% quartz glass) was used for sampling. The chamber featured inlet and outlet ports at its base, with one port left open to maintain stable internal pressure. It was positioned 20 cm beneath the LED light source.

#### Air supply and purification

Ambient air was drawn by a calibrated pump (SB-948, Beijing Institute of Labor Protection Science) at a constant flow rate of 900 ml·min^−^^1^. The air stream was passed through a trapping column packed with molecular sieve and charcoal (Supelpure HC) to remove ambient VOCs and moisture, ensuring a clean, hydrocarbon-free supply before entering the chamber via Teflon tubing.

#### Sampling procedure

Volatiles emitted from enclosed leaves were drawn from the outlet port at 100 ml·min^−^^1^ using a portable mini-sampler (Beijing Institute of Labor Protection) and trapped onto a BVOC-specific adsorption tube (Tenax TA, Markes, UK). Each sampling event lasted 30 min. Daily blank samples (empty chamber) were collected under identical conditions for background correction. Adsorption tubes were sealed and stored at 4 °C in the dark, with all samples analyzed within 48 h. The enclosed leaf area was determined by scanning after sampling for accurate emission-rate calculation.

#### BVOC analysis by TD-GC-MS

BVOCs were analyzed using thermal desorption-gas chromatography-mass spectrometry (TD-GC-MS) with a TD-100 unit (Markes, UK), GC-7890B, and MS-5977B (Agilent, USA). The detailed procedures and operational parameters are described in three steps below. (1) Thermal desorption: The adsorption tube was desorbed at 320 °C for 5 min. Volatile compounds were focused in a cold trap held at −10 °C, then rapidly heated for transfer to the GC. (2) Gas chromatographic separation: The separation was performed using a DB-Select 624 Ultra Inert capillary column (30 m × 250 μm × 1.4 μm) with helium carrier gas at a flow rate of 1.2 ml·min^−1^. The oven temperature program was as follows: 40 °C (hold 5 min), ramp up to 80 °C at 8 °C min^−1^ (hold 2 min), to 160 °C at 5 °C min^−1^ (hold 3 min), to 200 °C at 6 °C min^−1^ (hold 8 min), and post-run at 220 °C for 10 min. (3) MS spectrometric detection: An electron impact (EI) ion source was employed with the ion source temperature set at 230 °C and the quadrupole temperature at 150 °C. The mass scanning range was 45 to 350 m/z.

#### Compound identification and quantification

Qualitative identification was performed using MassHunter Qualitative Analysis software (Agilent, USA) with the NIST20.L mass spectral library (National Institute of Standards and Technology, USA) and chromatographic-grade standard alkane mixtures (Sigma). Identification was based on comparisons of mass spectra and retention indices (for details, see^[43]^).

Quantification used an external standard method with a certified gas mixture (Dalian Specialty Gases Co., China) containing 14 target compounds: methanol, acetaldehyde, isoprene, benzene, toluene, xylene, chlorobenzene, dichlorobenzene, acetonitrile, acrolein, crotonaldehyde, *α*-pinene, acetone, and 2-butanone. The original concentration of each compound was 1,000 ± 5 μmol·mol^−1^, v/v. Primary standards were dynamically diluted with high-purity N_2_ to generate a working concentration of 200 μmol·mol^−1^, v/v. To construct calibration curves, the diluted standard gas was sampled onto adsorption tubes at the same flow rate (100 ml·min^−1^) for different durations (10, 20, 30, and 40 min), yielding a series of absolute amounts (nmol) for each target compound. Calibration curves were established by plotting peak area against absolute amount (nmol), with *R*^2^ > 0.99 for all compounds. Emission rates were calculated based on the quantified amount, leaf area, sampling time, and flow rate. Daily blank samples (empty chamber) were analyzed under identical conditions, and their corresponding peak areas were subtracted from all sample measurements to correct for background contamination. All emission rates were expressed on a leaf area basis (nmol·m^−2^·s^−1^) to facilitate comparison with previous studies.

### Photosynthetic parameter measurements

Measurements of photosynthetic parameters were conducted using a portable photosynthesis system (GFS-300, WALZ, Germany). Healthy mature leaves were selected for measurements.

#### Baseline gas-exchange measurements

After 30 min of instrument warm-up and zeroing, baseline conditions were set: flow rate of 750 μmol·s^−1^, leaf chamber temperature of 28 °C, relative humidity of 60%−70%, CO_2_ concentration of 420 μmol·mol^−1^, and photosynthetic photon flux density (PPFD) of 800 μmol·m^−2^·s^−1^.

Leaves were equilibrated until stomatal conductance and net photosynthetic rate stabilized. The photosynthetic gas-exchange parameters recorded included net assimilation rate (*A*, μmol·m^−2^·s^−1^), transpiration rate (*E*, mmol·m^−2^·s^−1^), stomatal conductance (*G*s, mmol·m^−2^·s^−1^), and intercellular CO_2_ concentration (*C*_i_, μmol·mol^−1^). Water use efficiency (WUE) was calculated as WUE = *A*/*E*.

#### Light response curve measurements and parameter estimation

After baseline measurements, light response measurements were conducted. The PPFD (μmol·m^−2^·s^−1^) levels were set according to^[19]^, following the sequence: 800, 1,200, 1,600, 800, 400, 200, 100, 50, and 0. Each PPFD level was maintained for 3–5 min until a steady state. The light response curves were fitted using Ye Zipiao's modified rectangular hyperbola model^[48]^:

1 $ A_{}=\alpha _{\mathrm{p}}\frac{1-\beta _{\mathrm{p}}I}{1+\gamma _{\mathrm{p}}I}I-R_{\mathrm{d}} $

where, *A* is net assimilation rate, *I* is PPFD, *α*_p_ is the initial slope, *β*_p_ is the photoinhibition coefficient, *γ*_p_ is the light saturation coefficient, *R*_d_ is the dark respiration rate. The light saturation point *I*_sat_ and the maximum net assimilation rate *A*_max_ were calculated using Eqs. (2) and (3), respectively:

2 $ I_{\mathrm{sat}}=\frac{\sqrt{\left( \beta _{\mathrm{p}}+\gamma _{\mathrm{p}} \right) /\beta _{\mathrm{p}}}-1}{\gamma _{\mathrm{p}}} $

3 $ A_{\max}=\alpha _{\mathrm{p}}\left( \frac{\sqrt{\beta _{\mathrm{p}}+\gamma _{\mathrm{p}}}-\sqrt{\beta _{\mathrm{p}}}}{\gamma _{\mathrm{p}}} \right) ^2-R_{\mathrm{d}} $

#### CO_2_ response curve measurements and parameter estimation

After the base state stabilized and the stomata were fully open, CO_2_ concentrations were set as follows: 420, 200, 120, 60, 30, 15, 420, 600, 800, 1,200, and 1,600 μmol·mmol^−1^. Measurements were taken after stabilization at each concentration. *A*-*C*_i_ response biochemical model established by Farquhar et al.^[49]^ was employed for curve fitting. The model was expressed as follows:

4 $ A=\min \left\{ A_{\mathrm{c}},A_{\mathrm{j}} \right\} \left( 1-\frac{\varGamma ^*}{C_{\mathrm{i}}} \right) -R_{\mathrm{p}} $

where, *A* is net assimilation rate, *A*_c_ represents the CO_2_ assimilation rate limited by Rubisco activity, *A*_j_ indicates the CO_2_ assimilation rate constrained by the regeneration rate of ribulose-1,5-bisphosphate (RuBP), Г* refers to the CO_2_ compensation point in the absence of dark respiration, *C*_i_ stands for the intercellular CO_2_ concentration, and *R*_p_ represents the rate of respiration under light conditions. The mathematical expressions for *A*_c_ and *A*_j_ were given as follows:

5 $ A_{\mathrm{c}}=\frac{V_{\mathrm{c}\max}C_{\mathrm{i}}}{C_{\mathrm{i}}+K_{\mathrm{c}}\left( 1+O/K_{\mathrm{o}} \right)} $

6 $ A_{\mathrm{j}}=\frac{JC_{\mathrm{i}}}{4.5C_{\mathrm{i}}+10.5\varGamma ^*} $

where, *V*_cmax_ is the maximum rate of Rubisco carboxylation, *K*_c_ and *K*_o_ are the Michaelis–Menten constants for carboxylation and oxygenation, respectively, *O* is the oxygen partial pressure inside the stomata, and *J* is the electron transport rate at light saturation for RuBP regeneration.

#### Temperature response curve measurements and parameter estimation

After the base state stabilized and the stomata were fully open, temperature response curves were measured. Leaf chamber temperature (°C) was set in the sequence: 28, 23, 33, 38, 43, and 48. Each temperature was maintained for 5 min to ensure leaf temperature equilibrium. The curves were fitted using Ye-Zipiao's temperature response model (Eq. 7):

7 $ A=\alpha _{\mathrm{t}}\frac{1-\beta _{\mathrm{t}}t}{1+\gamma _{\mathrm{t}}t}t+R_{\mathrm{to}} $

where, *A* is net assimilation rate, *α*_t_ is the initial slope of the temperature response curve, *β*_t_ is the inhibition coefficient, *γ*_t_ is the saturation rate, *t* is the temperature, and *R*_to_ is the photorespiration rate. The optimum temperature (*T*_opt_) and the corresponding maximum net assimilation rate (*A*_tmax_) were calculated using Eqs. (8) and (9):

8 $ T_{\mathrm{opt}}=\frac{\sqrt{\left( \beta _{\mathrm{t}}+\gamma _{\mathrm{t}} \right) /\beta _{\mathrm{t}}}-1}{\gamma _{\mathrm{t}}} $

9 $ A_{\mathrm{t}\max }=\alpha _{\mathrm{t}}\left( \frac{\left( \sqrt{\beta _{\mathrm{t}}+\gamma _{\mathrm{t}}}-\sqrt{\beta _{\mathrm{t}}}\right)}{\gamma_{t}}\right)^2+R_{{\mathrm{to}}}  $

### Leaf trait measurements

After BVOCs were collected, leaf fresh weight (FW) was determined, and leaf area (LA) was scanned using an HP Scanjet 4890 scanner. Subsequently, the leaves were oven-dried at 80 °C until a constant weight was reached (difference between two consecutive measurements < 0.001 g), and dry weight (DW, g) was recorded. Leaf water content (LWC), specific leaf weight (SLW, defined as leaf dry weight per unit area), and specific leaf area (SLA, expressed as leaf area per unit dry weight) were then calculated using the following equations:

10 $ {\mathrm{LWC}}=100*({\mathrm{FW}}-{\mathrm{DW}})/{\mathrm{FW}} $

11 $ {\mathrm{SLW}}={\mathrm{DW}}/({\mathrm{LA}}\cdot 10^{-4}) $

12 $ \mathrm{SLA=LA/DW} $

where, LWC is expressed in %, FW is leaf fresh weight (g), DW is leaf dry weight (g), SLW is in g·m^−^^2^, LA is leaf area (cm^2^), and SLA is in cm^2^·g^−^^1^.

### Data processing and analysis

Microsoft Excel was used for the organization and preprocessing of raw data. The photosynthetic light response curves and temperature response curves were fitted using the Photosynthesis Model Simulation Software (PMSS) developed by Ye-Zhipiao to obtain the key parameters of the model. The photosynthetic CO_2_ response curves were fitted using the plantecophys package in R language to calculate relevant physiological and biochemical indicators.

Statistical analysis was performed using SPSS statistical software (v27): one-way or multi-way analysis of variance (ANOVA) and post hoc tests (Tukey's test) were used to evaluate significant differences among the seven species. For these comparisons, all six technical replicates per species (*n* = 6) were included as independent data points. The independent samples t-test was used to compare the mean differences between two BVOC chemotype groups (monoterpene-dominant, MTS, *n* = 4 species; isoprene-dominant, ISO, *n* = 3 species). For this between-group comparison, species averages (each derived from six technical replicates) were used as the unit of analysis, with degrees of freedom = 5. Spearman correlation analysis was carried out to explore the relationships among variables. All graphs were drawn using OriginPro 2024 software.

## Results

### Variations in leaf functional traits and growth habits among species

The seven species selected, representing different families and genera (Table 1), exhibited substantial variations in life form, leaf water content (LWC), specific leaf weight (SLW), specific leaf area (SLA), and ecological habit. In terms of life form, *Machilus leptophylla* was evergreen, *Ormosia hosiei* was semi-evergreen, and the remaining species were deciduous. With the exception of *Edgeworthia chrysantha*, which was a shrub, the other species are woody trees.

Considerable interspecific variation was observed in leaf functional traits. LWC ranged from 59% to 81%, SLW from 36.8 to 95.1 g·m^−2^, and SLA from 105 to 255 cm^2^·g^−1^. Among these, the shrub *E. chrysantha* exhibited the highest LWC (81.7%) and the lowest SLW (39.7 g·m^−2^), consistent with adaptation to semi-shaded and humid environments. The evergreen tree *M. leptophylla* displayed the highest SLW (95.1 g·m^−2^), indicative of denser leaf tissue and greater shade tolerance, which may reflect a carbon investment strategy favoring extended leaf lifespan under low light. By contrast, *W. sinensis* had the lowest SLW (36.8 g·m^−2^), suggesting relatively thin leaves that were consistent with its climbing habit and a strategy prioritizing rapid light capture and high photosynthetic turnover via expanded leaf area and reduced leaf mass.

### BVOC emission characteristics and patterns

**Figure 1:**
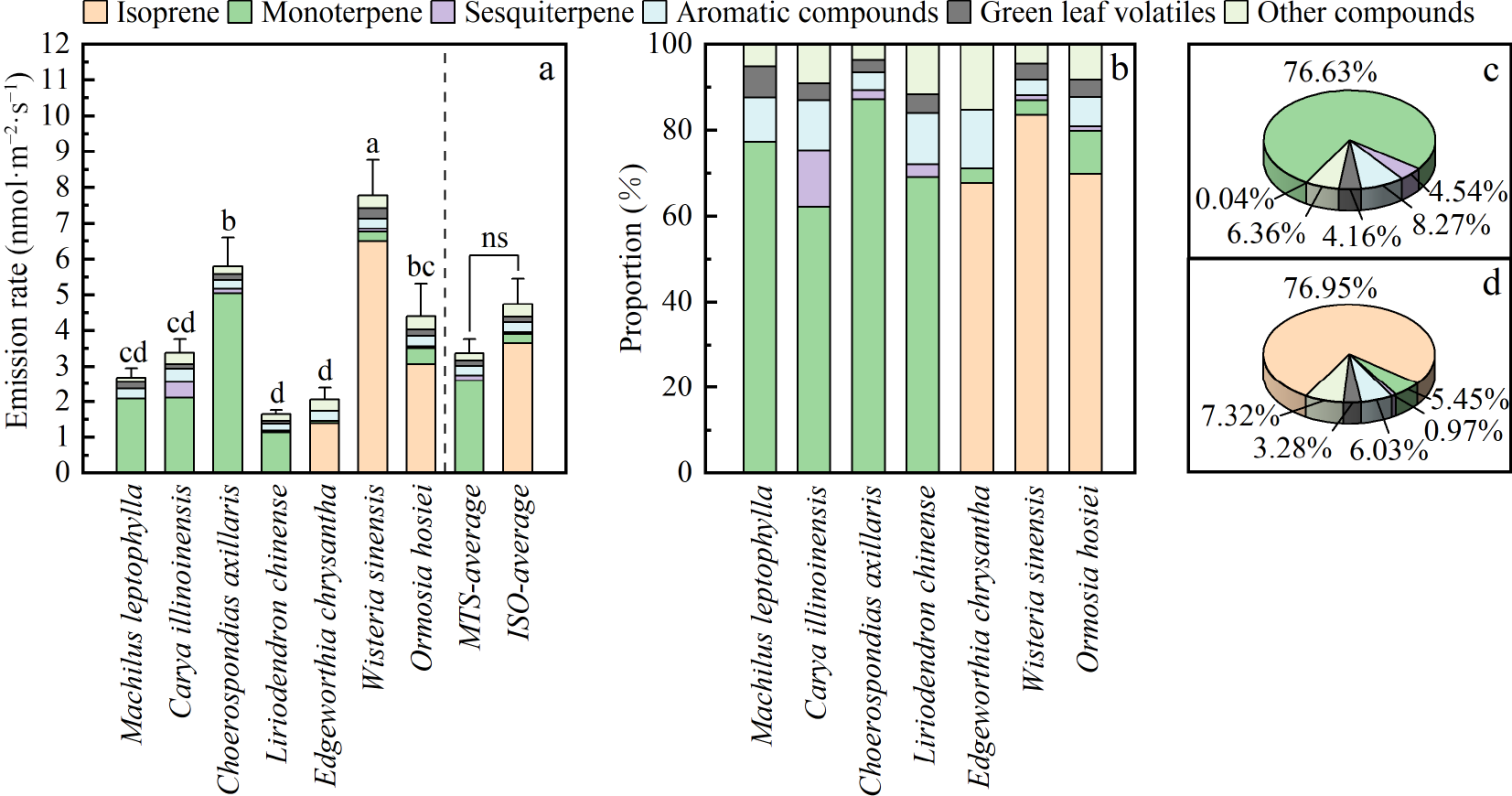
BVOC emission profiles of seven plant species. (a) Total BVOC emission rates. (b) Relative compositional proportion of BVOCs for each species. (c) Compositional proportion of isoprene-dominant emitters (ISO). (d) Compositional proportion of monoterpene-dominant emitters (MTS). BVOC emission rates (nmol·m^−2^·s^−1^) for individual compounds and total emissions are provided in Supplementary Table S1. Different lowercase letters indicate significant differences among species (Tukey's test, *p* < 0.05). 'ns' denotes no significant difference between ISO and MTS groups (independent samples *t*-test, *p* > 0.05). Values are mean ± SE (*n* = 6).

The seven plant species exhibited marked divergence in BVOC emission profiles (detailed in Supplementary Table S1, with key data summarized in Fig. 1). Based on the dominant compounds, the species were grouped into two dominant types: a monoterpene-dominant type (MTS, monoterpenes > 60% of total emissions) and an isoprene-dominant type (ISO, isoprene > 70%). Green leaf volatiles and aromatic compounds were emitted at low rates by both types, though their compositional profiles differed, suggesting potential divergence in metabolic pathways and ecological function.

The MTS species, *Machilus leptophylla*, *Carya illinoinensis*, *Choerospondias axillaris*, and *Liriodendron chinense* emitted primarily *α*-pinene, *β*-phellandrene, and *β*-pinene. Among these, *C. axillaris* was notable for its high emissions of *β*-ocimene and for having a significantly higher total BVOCs emission rate than other monoterpene emitters. The ISO species included *Edgeworthia chrysantha*, *Wisteria sinensis*, and *Ormosia hosiei*. *W. sinensis* exhibited the highest isoprene emission rate at 6.487 nmol·m^−2^·s^−1^, which was significantly greater than that of *E. chrysantha* and *O. hosiei*.

Although the mean total BVOC emission rate was higher in isoprene-dominant species than in monoterpene-dominant ones, the difference was not statistically significant.

### Photosynthetic response characteristics

**Figure 2:**
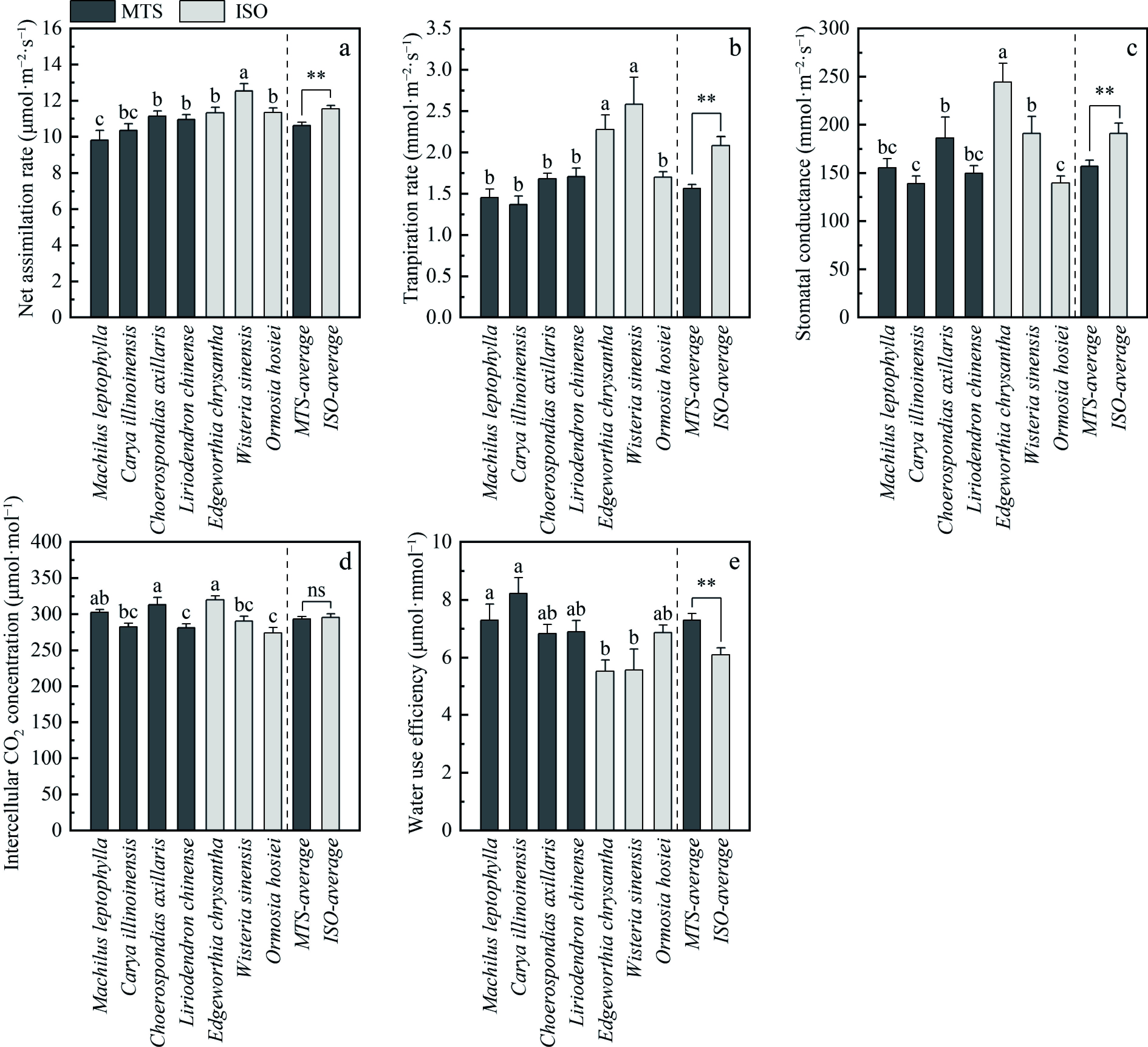
Photosynthetic parameters under baseline conditions (PPFD 800 μmol·m^−2^·s^−1^, CO_2_ 420 μmol·mol^−1^, 28 °C). MTS-average and ISO-average denote pooled values for monoterpene-dominant and isoprene-dominant types, respectively. Lowercase letters indicate significant differences among the seven species (Tukey's test, *p* < 0.05). Asterisks indicate significant differences between MTS and ISO groups (* *p* < 0.05, ** *p* < 0.01; independent samples *t*-test); 'ns' indicates no significant difference. Values are mean ± standard error (SE) (*n* = 6).

Under baseline conditions (PPFD 800 μmol·m^−2^·s^−1^, CO_2_ 420 μmol·mol^−1^, and 28 °C), significant interspecific differences and emission-type-specific patterns were observed in net assimilation rate (*A*), transpiration rate (*E*), stomatal conductance (*G*s), intercellular CO_2_ concentration (*C*_i_), and water use efficiency (WUE) (Fig. 2). ISO species (particularly *W. sinensis*) exhibited higher *A*, *E*, and *G*s, whereas MTS species (such as *Machilus leptophylla* and *Carya illinoinensis*) showed higher WUE, suggestive of a more conservative water use strategy. The absence of significant differences in *C*_i_ between the two groups suggested that divergence in stomatal behavior primarily underlies the differences in transpiration and water use efficiency.

**Figure 3:**
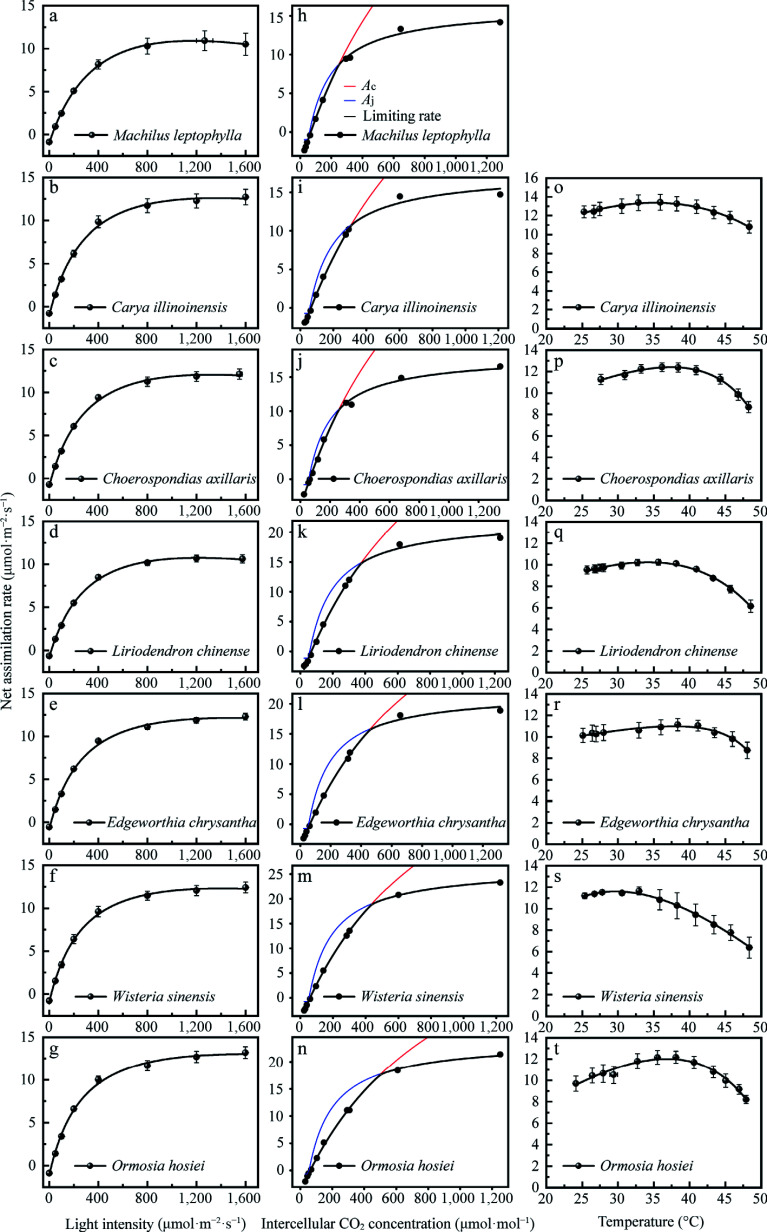
Photosynthetic response curves. (a)−(g) Light response curves (*A*-L). (h)−(n) CO_2_ response curves (*A*-*C*_i_). (o)−(t) Temperature response curves (*A*-T). *A*, net assimilation rate. *A*_c_ and *A*_j_ donate Rubisco activity-limited and RuBP regeneration-limited stages, respectively, derived from *A*-*C*_i_ curves. Curve fitting statistics are provided in Table 2 (*R*^2^ > 0.98, *p* < 0.05 for all fits).

Light response curves were fitted using the modified rectangular hyperbola model^[48]^. The model fitted all species well, with coefficient of determination (*R*^2^) ranging from 0.995 to 0.998 across the seven species (Table 2; Fig. 3). While no significant differences were found in maximum net assimilation rate (*A*_max_) among species, the light saturation point (*I*_sat_) was highest in *O. hosiei* (1,877 μmol·m^−2^·s^−1^), and the light compensation point (*I*_c_) was lowest in *E. chrysantha* (14.4 μmol·m^−2^·s^−1^). The lowest apparent quantum efficiency (*α*_p_) was observed in *M. leptophylla* (Fig. 3; Table 2).

**Table 2:** Photosynthetic parameters derived from the response curves to light (*A*-L), CO_2_ (*A*-*C*_i_) and temperature (*A*-*T*.) in seven woody species.

	Species	*Machilus leptophylla*	*Carya* *illinoinensis*	*Choerospondias* *axillaris*	*Liriodendron* *chinense*	*Edgeworthia* *chrysantha*	*Wisteria* *sinensis*	*Ormosia* *hosiei*
Fitting parameters of the light response curve	*A*_max_	11.118 ± 1.156a	12.61 ± 0.876a	12.117 ± 0.602a	10.85 ± 0.381a	12.154 ± 0.378a	12.333 ± 0.592a	13.1 ± 0.691a
	*I*_sat_	1,309.962 ± 67.986b	1,351.746 ± 28.021b	1,325.645 ± 67.633b	1,269.557 ± 82.395b	1,474.862 ± 47.225b	1,429.707 ± 116.537b	1,877.378 ± 166.234a
	*I*_c_	23.599 ± 2.874a	18.335 ± 0.856a	17.051 ± 1.956a	16.578 ± 2.646a	14.387 ± 1.131a	16.335 ± 4.216a	18.709 ± 4.339a
	*α*_p_	0.046 ± 0.002c	0.058 ± 0.005ab	0.056 ± 0.004abc	0.05 ± 0.003bc	0.057 ± 0.002ab	0.06 ± 0.006ab	0.066 ± 0.004a
	*R*_d_	1.031 ± 0.143a	1.005 ± 0.097a	0.895 ± 0.095a	0.776 ± 0.124a	0.784 ± 0.063a	0.932 ± 0.233a	1.113 ± 0.208a
	*R* ^2^	0.998	0.997	0.998	0.998	0.997	0.998	0.995
Fitting parameters of CO_2_ response curve	*V*_cmax_	57.66 ± 4.595a	60.462 ± 4.331a	65.766 ± 5.802a	67.173 ± 3.386a	56.394 ± 2.309a	67.213 ± 1.684a	62.018 ± 4.381a
	*J*_max_	75.331 ± 8.063e	81.705 ± 5.382de	87.955 ± 4.147cde	110.347 ± 8.418abc	103.649 ± 6.705bcd	130.073 ± 6.155a	117.24 ± 8.541ab
	*R*_p_	0.99 ± 0.111ab	0.798 ± 0.114ab	1.134 ± 0.195ab	1.328 ± 0.305a	0.714 ± 0.073b	0.74 ± 0.042ab	0.778 ± 0.109ab
	Γ*	68.527 ± 3.059ab	64.978 ± 2.238ab	67.686 ± 3.06ab	70.201 ± 4.286a	62.787 ± 1.173ab	59.053 ± 0.322b	63.008 ± 2.015ab
	*J*_max_ */V*_cmax_	1.299 ± 0.061b	1.366 ± 0.082b	1.393 ± 0.139b	1.637 ± 0.073ab	1.828 ± 0.055a	1.94 ± 0.123a	1.941 ± 0.186a
	*R* ^2^	0.996	0.995	0.989	0.997	0.997	0.999	0.997
Fitting parameters of temperature response curve	*α*_t_		0.594 ± 0.214b	0.364 ± 0.054b	0.407 ± 0.053b	0.473 ± 0.12b	1.403 ± 0.872a	0.493 ± 0.064b
	*A*_tmax_		13.359 ± 0.815a	12.465 ± 0.411ab	10.262 ± 0.285c	10.963 ± 0.662bc	11.868 ± 0.15abc	12.129 ± 0.626abc
	*T*_opt_		35.2 ± 0.43a	37.086 ± 0.965a	34.39 ± 0.85a	37.593 ± 1.078a	31.024 ± 1.89b	37.282 ± 0.636a
	*R* ^2^		0.985	0.988	0.995	0.972	0.975	0.967
Note: abbreviations: *A*_max_: maximum net assimilation rate (μmol·m^−2^·s^−1^); *I*_sat_: light saturation point (μmol·m^−2^·s^−1^); *I*_c_: light compensation point (μmol·m^−2^·s^−1^); *α*_p_: initial quantum efficiency (μmol CO_2_ μmol^–1^ photons); *R*_d_: dark respiration rate (μmol·m^-2^·s^−1^); *V*_cmax_: maximum carboxylation rate of Rubisco enzyme (μmol·m^−2^·s^−1^); *J*_max_: maximum electron transfer rate (μmol·m^−2^·s^−1^); *R*_p_: respiratory rate under light (μmol·m^−2^·s^−1^); Γ*: CO_2_ compensation point (μmol·mol^−1^); *J*_max_*/V*_cmax_: the ratio of *J*_max_ to *V*_cmax_ (μmol electrons [μmol CO_2_]^−1^); *α*_t_: initial slope (μmol·m^−2^·s^−1^); *A*_tmax_: maximum net assimilation rate in temperature response curve (μmol·m^−2^·s^−1^); *T*_opt_: optimal temperature (°C) corresponding to maxima net assimilation rate; *R*^2^: fitting coefficient. Values are mean ± SE (*n* = 6). Different letters within a row indicate significant differences (LSD test, *p* < 0.05).								

CO_2_ response curves were fitted using Farquhar's biochemical model^[49]^. The model provided an excellent fit for all species, with *R*^2^ values ranging from 0.989 to 0.997 (Table 2; Fig. 3). Among the fitted parameters, the maximum carboxylation rate (*V*_cmax_) did not differ significantly among species, whereas the maximum electron transport rate (*J*_max_) was highest in *W. sinensis* and lowest in *M. leptophylla*. The higher *J*_max_/*V*_cmax_ ratio observed in ISO species suggested a greater capacity for photosynthetic electron transport. Overall, isoprene emitters exhibited higher *J*_max_ and *J*_max_/*V*_cmax_ values, but lower respiration in light (*R*_p_) and CO_2_ compensation point (Γ*), compared to monoterpene emitters.

Temperature response curves were fitted using the temperature response model^[48]^. The model provided a good fit for all species, with *R*^2^ values ranging from 0.967 to 0.995 (Table 2; Fig. 3). The optimal photosynthetic temperature (*T*_opt_) ranged from 31.024 to 37.593 °C. *C. illinoinensis* and *W. sinensis* exhibited higher *T*_opt_ values, while *M. leptophylla* showed the lowest, indicating higher sensitivity to elevated temperature. The maximum net assimilation rate at *T*_opt_ (*A*_tmax_) was highest in *C. illinoinensis* and relatively low in *Liriodendron chinense* and *E. chrysantha*.

These results reveal interspecific differentiation and emission-type-specific patterns in photosynthetic traits, reflecting distinct photosynthetic adaptation strategies associated with different BVOC chemotypes.

### Correlation analysis among different trait parameters

**Figure 4:**
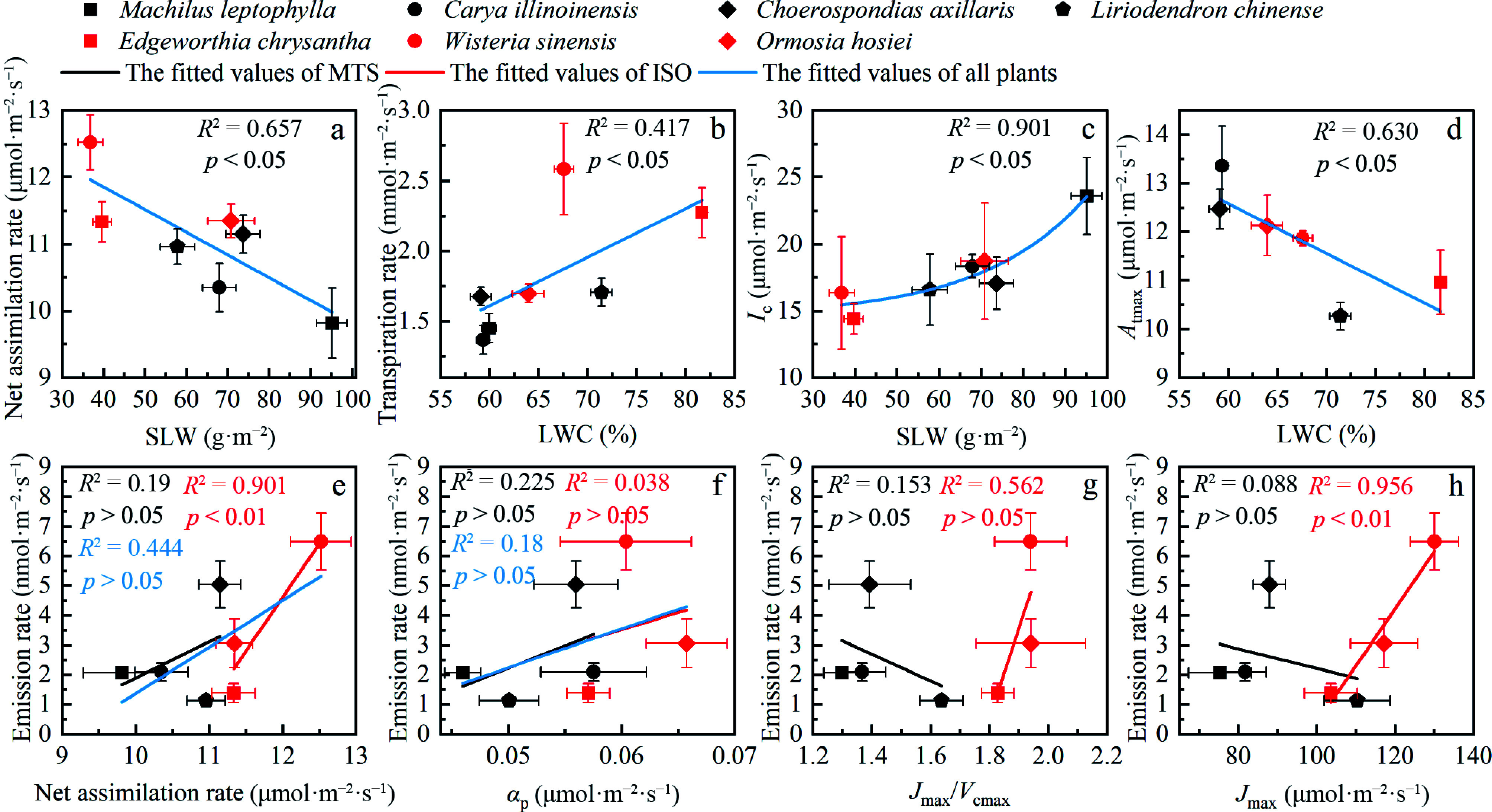
Correlations among leaf traits, photosynthetic parameters, and BVOC emissions. (a)–(d) Leaf traits vs photosynthetic parameters; (e)–(h) BVOC emissions vs photosynthetic parameters. Correlation coefficients (*R*^2^) and significance levels are shown for each relationship (ns, *p* > 0.05; * *p* < 0.05; ** *p* < 0.01). Abbreviations: SLW, specific leaf weight; LWC, leaf water content; other parameters defined in Table 2.

Spearman correlation and regression analyses (Fig. 4) revealed systematic relationships between BVOC emission rates and photosynthetic parameters and leaf structural traits, with distinct patterns observed between MTS and ISO plants.

Specific leaf weight (SLW) was strongly negatively correlated with net assimilation rate (*A*) under baseline conditions (*R*^2^ = 0.657, *p* < 0.05) and strongly positively correlated with the light compensation point (*I*_c_) (*R*^2^ = 0.901, *p* < 0.05). Leaf water content (LWC) was positively correlated with transpiration rate (*E*) (*R*^2^ = 0.417, *p* < 0.05) and negatively correlated with the net photosynthetic rate at optimum temperature (*A*_tmax_) (*R*^2^ = 0.562, *p* > 0.05).

Isoprene emission was strongly positively correlated with net assimilation rate (*R*^2^ = 0.901, *p* < 0.01), *J*_max_ (*R*^2^ = 0.956, *p* < 0.01). These correlations were stronger for isoprene than for monoterpenes, with monoterpene emission showing no significant correlation with these parameters (*p* > 0.05 for all). Moreover, isoprene emission showed a stronger correlation with *J*_max_ than with *J*_max_/*V*_cmax_, suggesting that its emission is more closely linked to photosynthetic electron transport capacity. These findings indicated divergent regulatory mechanisms between monoterpene- and ISO-type plants.

**Figure 5:**
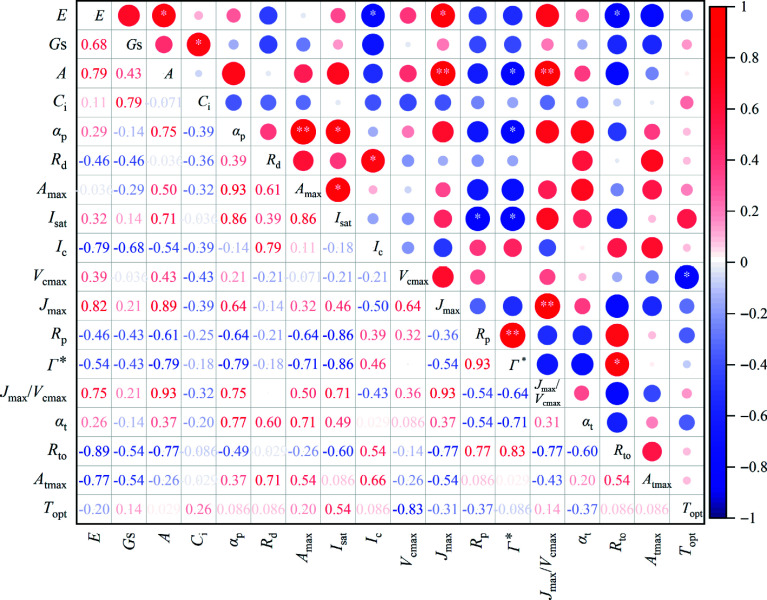
Spearman correlation matrix of photosynthetic parameters. *E*, Transpiration rate, *G*s: Stomatal conductance, *A*: Net assimilation rate, *C*_i_: Intercellular CO_2_ concentration; other parameters (*α*_p_, *R*_d_, *A*_max_, *I*_sat_, *I*_c_, *V*_cmax_, *J*_max_, *R*_p_, Γ*, *J*_max_*/V*_cmax_, *α*_t_, *A*_tmax_, and *T*_opt_) defined in Table 2. Color scale indicates correlation coefficient (*r*). * *p* < 0.05, ** *p* < 0.01.

Furthermore, significant intrinsic correlations were identified among photosynthetic parameters (Fig. 5). Net photosynthetic rate was strongly positively correlated with stomatal conductance and transpiration rate (*r* = 0.68); light saturation point (*I*_sat_) was significantly correlated with maximum net photosynthetic rate (*A*_max_) (*r* = 0.86); light compensation point (*I*_c_) was negatively correlated with apparent quantum efficiency (*α*_p_) (*r* = −0.79); and CO_2_ compensation point (Γ*) was negatively correlated with the *J*_max_/*V*_cmax_ ratio (*r* = −0.64). Together, these relationships reflect key trade-offs and synergies within the photosynthetic apparatus pertaining to light capture, carbon assimilation, and water-use strategies.

## Discussion

This study systematically examined variations and synergies in BVOC emissions, photosynthetic characteristics, and leaf structural traits among seven common subtropical woody plants. By deliberately focusing on moderate emitters from diverse lineages, we aimed to elucidate the intrinsic 'structure–function–volatile' relationships without the potential confounding effects of hyper-emission, which can disproportionately dominate both metabolic budgets and functional interpretations. The results revealed significant functional differentiation and intrinsic correlations among BVOC chemotypes, photosynthetic energy-use strategies, and leaf structural traits. The following sections integrate and discuss these findings from three perspectives: species-specific metabolic strategies, the driving role of photosynthetic energy and carbon supply, and the long-term evolutionary adaptations underpinning these coordinated traits.

### Species-specific BVOC emission profiles and metabolic differentiation

The seven studied species, representing seven different genera and diverse life forms (evergreen/deciduous trees, shrubs, and lianas), exhibited pronounced differences in BVOC composition and emission rates (Supplementary Table S1; Table 1). Crucially, this chemical divergence was not random but aligned with and reinforced their positions along the Leaf Economics Spectrum (LES) and within the fundamental growth-defense trade-off, reflecting evolutionary adaptations to distinct ecological niches^[8,18,20,50]^.

Monoterpene-dominant emitters (MTS: *M. leptophylla*, *C. illinoinensis*, *C. axillaris*, and *L. chinense*) exhibited monoterpene proportions of 60%–87%, with *α*-pinene, *β*-pinene, and D-limonene as major constituents. These compounds are well-documented for their roles in direct insect resistance, antimicrobial activity, and plant-plant signaling^[8,50]^. This strategy epitomizes the 'Conservative-Storage' syndrome^[5,50]^, characterized by high investment in stored volatiles as part of a broader resource allocation toward durable defense and long-lived leaves (high SLW, Table 1)^[51]^.

Among the MTS species, *M. leptophylla* represented the most extreme expression of this conservative strategy: it was an evergreen tree with the highest SLW (95.1 g·m^−2^) and the lowest leaf water content (59%) among all studied species (Table 1), reflecting dense leaf tissues optimized for long-term carbon retention and stress resistance. Its monoterpene profile was dominated by *α*-pinene and *β*-pinene, with minimal sesquiterpene diversity, suggesting a streamlined, constitutive chemical defense arsenal typical of shade-tolerant, slow-growing evergreens^[52]^. In contrast, *C. illinoinensis* produced a diverse array of sesquiterpenes (e.g., caryophyllene, germacrene D, and *β*-cadinene), which typically function in long-term defense due to their lower volatility and higher molecular stability^[17]^. *L. chinense*, while sharing the basic MTS profile, exhibited relatively higher light respiration rates (Table 2), suggesting a greater diversion of photosynthetic carbon to maintenance processes that might compete with *de novo* monoterpene synthesis.

A notable specialization within the MTS syndrome was observed in *C. axillaris*, which emitted high levels of the light-dependent monoterpene *β*-ocimene (constituting 75% of its total monoterpenes). Unlike the stored monoterpenes typical of MTS species, *β*-ocimene biosynthesis relied on recently fixed photosynthetic carbon via the plastidal MEP pathway, and its synthase shared high sequence homology with isoprene synthase, suggesting a common evolutionary origin. Ecologically, *β*-ocimene acted as a herbivore-induced plant volatile (HIPV) that can directly activate plant defense, prime neighboring plants, attract natural enemies, and facilitate pollinator attraction^[7,53,54]^. Within the high-light, high-temperature canopy environment where *C. axillaris* thrived, rapid *de novo* synthesis of *β*-ocimene allowed for immediate stress signaling without requiring constitutive storage. Thus, *C. axillaris* represented an intermediate strategy within the MTS continuum: it retained the core 'conservative' leaf traits (high SLW, Table 1) and stored a monoterpene pool, but had evolved a light-triggered, rapidly inducible *β*-ocimene channel for dynamic ecological communication. This flexibility suggested that the ISO–MTS distinction represented endpoints along a continuum rather than a strict dichotomy, illustrating that volatile strategies were better understood as a spectrum shaped by species-specific niches and phylogenetic history.

Additionally, certain sesquiterpenes were detected in both *C*. *illinoinensis* (e.g., caryophyllene, *trans*-*α*-bergamotene, [Z,Z]-*α*-farnesene, germacrene D, and *β*-cadinene) and *C*. *axillaris* (e.g., [E]-*β*-farnesene and caryophyllene), which typically functioned in long-term defense due to their higher molecular stability and lower volatility^[17]^. Recent breakthroughs further highlighted that sesquiterpenes like (−)-germacrene D were specifically perceived by the karrikin signaling receptor KAI2, triggering developmental cascades critical for reproductive organ maturation^[55]^, implying unrecognized ecological functions of these emissions in our study species.

Isoprene-dominant emitters (ISO: *E. chrysantha*, *W. sinensis*, *O. hosiei*) demonstrated high isoprene emission capacities, aligning with the 'Acquisitive-Overflow' syndrome^[33,50]^. This pattern was consistent with a strategy characterized by rapid carbon acquisition and growth, as reflected in their lower SLW and higher SLA (Table 1)^[56]^. Isoprene emission functioned as a real-time metabolic sink for excess photosynthetic energy, crucial for plants maintaining high metabolic flux^[20,57]^. The shared high isoprene emission in *W. sinensis* and *O. hosiei* suggested a phylogenetically conserved trait, consistent with observations that Fabaceae trees in subtropical regions frequently adopt isoprene emission-type strategies^[7]^. *W. sinensis*, as a liana, also exhibited relatively high emissions of GLVs, reflecting its biotic stress protection needs under high growth rates^[30,58]^. *O. hosiei*, as a semi-evergreen tree, retained some capacity for monoterpene emission, suggesting greater flexibility in chemical response to seasonal changes in temperature and herbivory pressure^[34]^.

*E. chrysantha* presents a specialized case, emitting almost exclusively isoprene with an elevated *J*_max_/*V*_cmax_ ratio, demonstrating that the ISO syndrome is not exclusive to Fabaceae. We acknowledge that two of the three ISO species belong to Fabaceae, whose high nitrogen fixation capacity could partly contribute to the observed physiological differences between ISO and MTS groups. However, the similar high-isoprene, high- *J*_max_/*V*_cmax_ traits in *E. chrysantha* suggest the acquisitive-overflow syndrome is not restricted to Fabaceae. Given the limited sample size (*n* = 7), we cannot fully disentangle phylogenetic effects from emission strategy; future studies with larger species sets and phylogenetic independent contrasts are needed. This point is further discussed in the context of the strategy continuum below.

Critically, these divergent BVOC profiles are integral components of coherent functional syndromes^[33,50,59]^. MTS chemistry is embedded within a 'slow-return' strategy emphasizing preemptive investment in defense and structure, often accompanied by other costly, stored secondary metabolites (e.g., phenolics, alkaloids)^[52,60]^. In contrast, ISO chemistry was a hallmark of a 'fast-return' strategy that minimized fixed defense investment, favoring dynamic, energy-dependent protection and resulting in generally lower concentrations of other constitutive secondary metabolites^[32,61]^. Thus, BVOC chemotypes were robust indicators of a plant's fundamental position on the axis of resource acquisition vs. conservation.

It should be noted that the monoterpene and sesquiterpene emission rates reported here, measured from excised branches, might overestimate constitutive baseline levels for species with pressurized storage structures due to potential release upon cutting. Nonetheless, while excision may influence absolute emission rates, the relative relationships remained robust between the MTS emission profile^[62]^, representing a storage-based strategy, and its associated suite of conservative leaf traits^[43]^. These integrated relationships were consistent with the 'Conservative-Storage' syndrome as a coherent evolutionary strategy.

### Driving role of photosynthetic carbon metabolism and energy supply

#### Coupling of BVOC emissions with instantaneous photosynthesis: ISO vs. MTS types

BVOC synthesis is intrinsically linked to photosynthetic carbon and energy metabolism. The MEP pathway, localized in chloroplasts, serves as a critical bridge between photosynthesis and isoprene/monoterpene formation, directly generating DMAPP and IPP, the precursors for isoprene and most monoterpenes^[1,20,26,63]^.

Our data revealed a fundamental dichotomy in this coupling between the two chemotypes. ISO species showed higher net assimilation rates than monoterpene emitters (Fig. 2), and isoprene emission correlated strongly with instantaneous photosynthetic rate (Fig. 4). This tight linkage indicated that isoprene synthesis likely acted as a real-time metabolic sink, directly dependent on substrates and energy (ATP, NADPH) derived from concurrent light reactions and carbon fixation^[2,64]^. Under high light, ISO species possessed high light saturation points that likely channeled excess photosynthetic electrons and carbon into the MEP pathway^[2,8,50,65]^. This process was widely proposed to function as an ‘overflow metabolism' that helped mitigate photodamage. Recent evidence suggested that the photoprotective effect was achieved primarily through the stabilization of chloroplast membranes under thermal and oxidative stress, rather than via direct ROS scavenging^[21,66]^.

In contrast, monoterpene emissions were weakly correlated with instantaneous photosynthetic rates. This decoupling stemmed from a distinct storage-based strategy^[64]^. Monoterpenes are often synthesized during periods of high photosynthetic activity and sequestered in specialized structures (e.g., resin ducts, glandular trichomes), with emission being induced later by external stimuli such as herbivory or mechanical damage, rather than by real-time photosynthetic flux^[5,62]^. This represented a 'pre-formed defense' strategy, prioritizing resource allocation to durable chemical pools over continuous, energy-intensive synthesis.

#### Photosynthetic electron transport and energy shunting drive divergent BVOC emission patterns in ISO vs. MTS types

The divergence in BVOC emission strategies appeared to be largely associated with how plants manage photosynthetic electron flow and energy partitioning. ISO plants exhibited higher *J*_max_ and elevated *J*_max_/*V*_cmax_ ratios compared to MTS-type emitters (Fig. 2). An increased *J*_max_ reflected a greater capacity for light harvesting, whereas a higher *J*_max_/*V*_cmax_ ratio indicates that the capacity for light harvesting and electron transport (light reaction) could exceed the downstream demand for carbon fixation by the Calvin cycle (dark reactions), particularly under high light. This imbalance created a surplus of reducing equivalents (NADPH) and ATP^[2,8,64]^.

Our data strongly suggested that isoprene synthesis acted as a critical electron shunt under these conditions. The strong positive correlation between isoprene emission and *J*_max_ (Fig. 4e), and its direct dependence on electron transport capacity suggested that isoprene biosynthesis might consume excess reductants through the MEP pathway, thereby mitigating electron overflow and protecting photosystems from photoinhibition^[8,67,68]^.

In contrast, monoterpene emitters showed lower *J*_max_/*V*_cmax_ ratios but higher light respiration rates (*R*_p_, Table 2). This physiological profile indicated a strategy geared toward carbon conservation and efficient resource investment. Higher *R*_p_ suggested significant diversion of photosynthetic products into photorespiration and maintenance processes, which might compete with the MEP pathway for carbon skeletons and energy, limiting immediate *de novo* terpene synthesis^[69]^. This further explained the weaker link between monoterpene emission and instantaneous photosynthetic parameters (Fig. 4), emphasizing a reliance on mobilized storage rather than real-time production^[5]^.

Collectively, these findings supported the existence of two dominant adaptive syndromes linked to photosynthetic energy management. ISO-type plants prioritized rapid light capture and carbon assimilation, utilizing isoprene synthesis as a dynamic electron shunt to manage photosynthetic overflow and conferred immediate stress tolerance^[32,68]^. In contrast, the prevailing MTS-type strategy emphasized carbon economy and investment in storage-based defenses, uncoupling defense deployment from instantaneous metabolic flux to ensure persistent protection^[5]^.

#### Variations within the ISO–MTS continuum refine a spectrum of adaptive strategies

The ISO–MTS framework delineated dominant adaptive peaks in plant volatile strategies. However, the precise position of a species along this strategic continuum was shaped by the integration of its ecological context, phylogenetic history, and multifunctional traits. The cases of *C. axillaris* and *E. chrysantha* refined the simple dichotomy and demonstrated that these strategies represented a physiologically plastic spectrum of photosynthetic energy allocation and defense investment.

*C. axillaris*, classified within the MTS group due to its high monoterpene proportion, exemplified a hybrid strategy. Unlike typical MTS emitters, it lacked typical storage structures and emitted high levels of *β*-ocimene, a monoterpene known for light-dependent *de novo* synthesis^[5,70]^. This photosynthetically coupled volatile production reflected a physiological linkage characteristic of the ISO syndrome, likely reflecting adaptation to its high-light, canopy-gap niche where rapid, inducible aerial signaling for pollinator attraction or indirect defense was advantageous^[7,53]^. Thus, *C. axillaris* occupied an intermediate position, blending storage-oriented defense with a photosynthetically coupled, rapid-response volatile channel.

Conversely, *E. chrysantha*, a strong isoprene emitter representing the ISO type, exhibits a more nuanced energy allocation pattern. Despite possessing an acquisitive physiological base (high *J*_max_/*V*_cmax_) conducive to channeling excess energy into isoprene, its concurrently high *R*_p_, presence of glandular structures, and the accumulation of flavonoids suggest that a substantial portion of the excess energy is instead allocated to secondary metabolism and defense, rather than being fully committed to isoprene production^[71]^. These traits suggested significant resource partitioning toward photorespiratory maintenance and non-volatile chemical defenses. This pattern implied a strategic trade-off in which maximizing isoprene yield was secondary to maintaining a diversified defense portfolio, likely an adaptation to an ecological context where multiple abiotic and biotic stresses co-occur.

These variations did not invalidate the core ISO–MTS framework but confirmed it as a physiologically plastic continuum. They highlighted that a plant's volatile emission strategy was an integrated component of its overall ecological strategy, subject to fine-tuning in response to habitat-specific selection pressures. Intra-group variations, such as the elevated *R*_p_ in *L. chinense* potentially limiting monoterpene synthesis, further illustrated the continuous nature of trait coordination within these broad syndromes.

### Coordinated associations among leaf traits, photosynthetic physiology, and BVOC emissions

**Figure 6:**
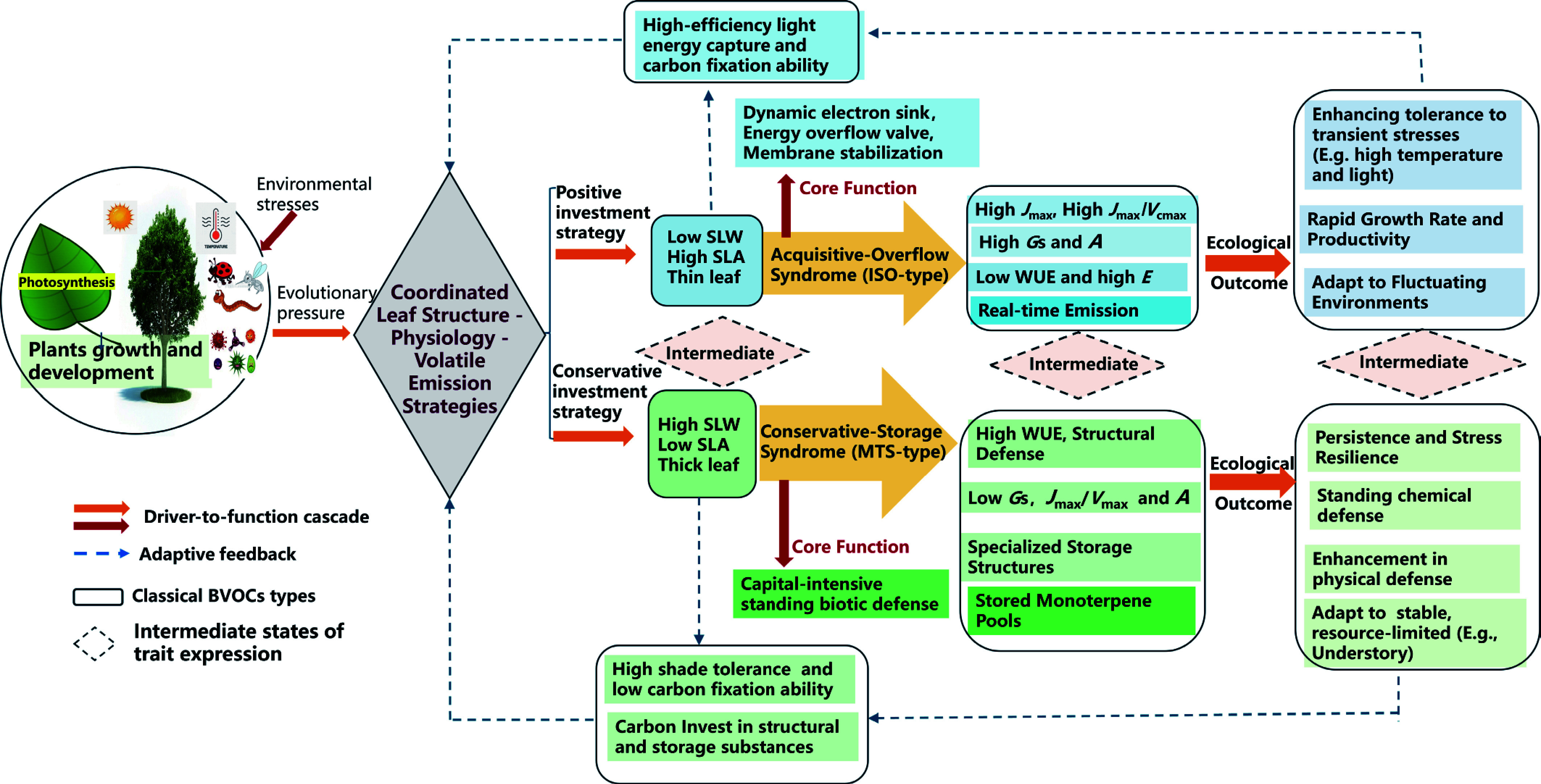
Conceptual model of structure–function–volatile syndromes in subtropical woody species. Solid arrows indicate environmental drivers; dotted arrows indicate functional feedbacks; dashed box represent intermediate trait states; and solid boxes represent classical ISO/MTS emission traits. Abbreviations: ISO, isoprene-dominant; MTS, monoterpene-dominant; others defined in Tables 1 and 2.

The integrated 'structure–function–volatile' patterns observed in this study (Fig. 6) were not merely correlative but represent evolutionary adaptations shaped by long-term natural selection^[50,72,73]^. They arose from divergent solutions to the universal growth-defense trade-off, as conceptualized within the Leaf Economics Spectrum (LES), wherein plants optimized the allocation of finite resources^[56,60,61]^.

#### The ISO-type adaptive syndrome

BVOC chemotypes appeared to reflect core ecological strategies. ISO species (e.g., *W. sinensis*, *E. chrysantha*) aligned with the 'fast-return' end of the LES. Their traits, including low specific leaf weight (SLW), high specific leaf area (SLA, expressed as leaf area per unit dry weight), high *J*_max_, and high stomatal conductance, were hallmarks of a strategy prioritizing rapid carbon acquisition and high metabolic flux^[50,56]^. This 'acquisitive' resource allocation minimized investment in long-lived structural components like cell walls, allowing greater allocation to the photosynthetic apparatus^[20,74]^. Consequently, these plants frequently faced a transient excess of photosynthetic energy (high *J*_max_/*V*_cmax_ ratio). The real-time, energy-dependent synthesis of isoprene acted as a dynamic 'metabolic overflow valve', consuming excess reductants and providing immediate membrane stabilization and thermotolerance^[21,32,66]^. This strategy was metabolically costly but allowed for rapid growth and is likely underpinned by a physiological state where growth-promoting signals were predominant^[61]^. It represented a high-investment, high-reward strategy advantageous in environments with abundant light and water but fluctuating conditions (e.g., forest edges, gaps). Accordingly, ISO-type leaves typically contain lower concentrations of other costly, stored secondary metabolites (e.g., complex phenolics).

#### The MTS-type adaptive syndrome

In contrast, MTS species align with the 'slow-return, conservative' end of the LES. Their high SLW, higher water-use efficiency (WUE), and lower *J*_max_/*V*_cmax_ ratio indicated substantial investment in durable leaf structures (e.g., thickened cell walls) and resource conservation^[51]^. This enhanced leaf longevity and stress resilience at the cost of maximum photosynthetic throughput. Within this framework, defense was achieved through pre-emptive investment in stored compounds in resource-limited or stable environments (e.g., forest understories). Monoterpenes were synthesized and sequestered in specialized structures (e.g., resin ducts), constituting a 'standing defense' pool^[5]^. This pool was often part of a broader defensive arsenal.

In our study, typical MTS species like *M. leptophylla* and *C. illinoinensis* not only possessed secretory structures for monoterpene storage but also likely accumulated non-volatile defensive compounds such as phenolics, reflecting a comprehensive, capital-intensive investment in durable leaf defense and extended leaf lifespan. Their core function involved preferential carbon investment into structural/storage substances and the synergistic enhancement of physical and chemical defense. This strategy uncoupled defense readiness from instantaneous photosynthetic yield, minimizing metabolic opportunity costs. It suggests a physiological bias towards defense-signaling pathways^[61]^ and was a bet-hedging strategy well-suited to stable, resource-limited, or herbivore-rich environments like forest understories. This suite of traits often co-occurred with other stored defenses (e.g., tannins, alkaloids), forming a diversified defense portfolio.

It was noteworthy that our measurements of these stored volatiles were conducted on excised branches, a standard approach for comparative physiology. For species with pressurized secretory structures (e.g., resin ducts), excision might potentially lead to an overestimation of constitutive baseline emissions by releasing stored pools. However, this potential methodological effect did not undermine the core finding of strong integration between the MTS emission strategy (whether measured as stored capacity or induced release) and conservative leaf traits (e.g., high SLW). These correlations robustly reflected the long-term, 'slow-return' investment syndrome. Future *in situ* studies would be valuable to precisely quantify emission fluxes from intact plants in their natural context.

#### Framework validation across a broader spectrum

Therefore, the ISO–MTS divergence in BVOC emissions was a core component of broader, genetically correlated trait syndromes arising from adaptive optimization to different environmental selection pressures^[60]^. While our study established strong phenotypic integration, the direction of causality was best interpreted through an evolutionary lens. Habitats favoring rapid growth and high productivity selected for the integrated 'Acquisitive-Overflow' syndrome, where isoprene emission was a co-adapted trait for managing energy surplus. Conversely, habitats favoring persistence under chronic stress selected for the 'Conservative-Storage' syndrome, where volatile storage was part of a durable, pre-formed defense package. Thus, the observed correlations emerged from divergent evolutionary optima. This framework posited that the demonstrated physiological linkages (e.g., high *J*_max_ driving isoprene synthesis) were the proximate mechanisms underlying ultimate evolutionary strategies shaped by environmental filtering. This provided a mechanistic, adaptive explanation for the diversity of plant volatile strategies and their embeddedness within the fundamental axes of plant ecological variation.

The framework also generates testable hypotheses. For example, manipulating photosynthetic electron flow could test whether *J*_max_ drives isoprene emission in ISO species (with negligible impact on stored pools in MTS species). Extending the framework to hyper-emitters and diverse biomes would clarify its generality. These are essential steps toward integrating functional traits into next-generation models.

### Limitations and future perspectives

While this study integrates BVOC emissions within the leaf economics and growth-defense trade-off framework, providing a mechanistic, trait-based understanding of plant volatile diversity, several limitations point to valuable future directions.

#### Phylogenetic limitations

We acknowledge that the current study includes only seven species, which precluded rigorous phylogenetic independent contrasts (PIC) analysis. Consequently, we could not fully separate the effects of shared ancestry from adaptive convergence in the observed trait correlations. The ISO group includes two Fabaceae species and one Thymelaeaceae species (*E. chrysantha*), and the similar physiological traits among them might partly reflect phylogenetic relatedness rather than solely the isoprene emission strategy. Future studies with a larger and phylogenetically more diverse species set, ideally enabling PIC analysis, are needed to strengthen causal inference regarding ISO–MTS syndromes.

#### Validating the framework across a broader taxonomic and ecological spectra

Our focused experimental design with seven subtropical species clearly delineated the core ISO and MTS syndromes, including intermediates such as *C. axillaris*. Future work should test this 'structure–function–volatile' framework across a wider taxonomic and functional range, including hyper-emitters (e.g., *Populus*, *Quercus*), temperate broadleaf trees (e.g., *Fagus*, *Acer*), tropical shrubs (e.g., *Piper*, *Psychotria*), herbaceous plants, and species from diverse biomes, to determine whether these syndromes represent universal adaptive peaks or are regionally filtered.

#### Bridging controlled physiology with in situ ecology

Measurements on excised branches under controlled conditions enabled precise physiological comparisons. To understand how these trait networks operate in natural settings, future *in situ* monitoring of intact plants is essential. This will reveal how whole-plant integration, diurnal cycles, and field conditions (e.g., drought, biotic interactions) modulate the proposed syndromes, thereby linking mechanisms to ecology.

#### Establishing causality and understanding temporal dynamics

This study provided a strong correlative framework. To establish causal links, manipulative experiments (e.g., ^13^C-labeling to trace carbon flux or genetic manipulation of isoprenoid synthases) are needed to test the functional role of specific volatiles within these strategies. Furthermore, the seasonal and phenological dynamics of these coordinated traits remain open questions. Investigating their responses to environmental manipulations (e.g., elevated CO_2_, warming, drought) would clarify the plasticity and stability of ISO and MTS strategies over time.

Additionally, while our use of excised branches under controlled conditions enabled precise physiological comparisons, future *in-situ* measurements on intact plants would help validate whether the observed trait-emission relationships accurately reflect constitutive emission patterns, particularly for storage-type monoterpene emitters.

Addressing these directions would refine the proposed framework and deepen the integration of plant ecophysiology with atmospheric science, ultimately improving predictions of ecosystem–climate feedbacks.

## Conclusions

This study provides evidence that leaf structural traits, photosynthetic physiology, and BVOC emissions are intrinsically linked, forming coherent 'structure–function–volatile' syndromes in subtropical woody plants. Specifically, isoprene-dominant (ISO) species exhibited higher photosynthetic rates, greater electron transport capacity, and elevated *J*_max_/*V*_cmax_ ratios, consistent with isoprene synthesis functioning as a photosynthetic overflow mechanism for dissipating excess reductants. Conversely, monoterpene-dominant (MTS) species displayed higher specific leaf weight (SLW) and relied on stored emissions, reflecting a conservative, defense-oriented strategy aligned with the 'slow-return' end of the Leaf Economics Spectrum. Together, these findings demonstrate that BVOC chemotypes are not metabolically arbitrary but represent integrated evolutionary solutions to the universal growth–defense trade-off. Consequently, the proposed 'Acquisitive-Overflow' (ISO) and 'Conservative-Storage' (MTS) syndromes offer a trait-based framework for predicting volatile diversity from readily measurable plant functional traits. Future research extending this framework to hyper-emitters and diverse biomes will clarify its generality. By embedding BVOC emissions within the Leaf Economics Spectrum, this study bridges plant ecophysiology with atmospheric science, providing a physiological basis for next-generation models that forecast biosphere–atmosphere feedbacks under global change.

## SUPPLEMENTARY DATA

Supplementary data to this article can be found online.

## Data Availability

The datasets generated during and/or analyzed during the current study are available from the corresponding author upon reasonable request.
