# Supplementary material for: Divergence in isoprenoid emissions among subtropical broadleaf trees: linking photosynthesis, leaf economics, and volatile defense for forest management
Source: For Res (Fayettev). 2026 Jul 29;6:e024. doi: 10.48130/forres-0026-0023 (PMC13447279; doi:10.48130/forres-0026-0023)
Supplement: Supplementary file 1 [file FR-2026-6-0023-S1.pdf]

Supplementary Table S1. BVOCs emission rates and composition for the seven species.

Classification	Name	Formula	*Machilus leptophylla*	*Carya illinoinensis*	*Choerospondias axillaris*	*Liriodendron chinense*	*Edgeworthia chrysantha*	*Wisteria sinensis*	*Ormosia hosiei*
			Emission rate (nmol•m^{-2}•s^{-1})	Emission rate (nmol•m^{-2}•s^{-1})	Emission rate (nmol•m^{-2}•s^{-1})	Emission rate (nmol•m^{-2}•s^{-1})	Emission rate (nmol•m^{-2}•s^{-1})	Emission rate (nmol•m^{-2}•s^{-1})	Emission rate (nmol•m^{-2}•s^{-1})
Isoprene	Isoprene	C_5H_8	0.002 ± 0c	0.002 ± 0.001c	0.001 ± 0c	0.001 ± 0.001c	1.379 ± 0.316c	6.487 ± 0.957a	3.061 ± 0.825b
Monoterpene	*α*-phellandrene	$C_{10}H_{16}$	0.076 ± 0.008b	0.09 ± 0.009a	–	0.049 ± 0.004c	–	–	–
	α-pinene	$C_{10}H_{16}$	0.342 ± 0.031a	0.109 ± 0.015bc	0.064 ± 0.006cd	0.147 ± 0.033b	–	–	0.048 ± 0.004d
	Camphene	$C_{10}H_{16}$	0.07 ± 0.009b	0.086 ± 0.008a	0.061 ± 0.006bc	0.049 ± 0.004c	–	–	–
	β-phellandrene	$C_{10}H_{16}$	0.516 ± 0.089a	0.738 ± 0.233a	–	0.067 ± 0.006b	–	–	–
	β-pinene	$C_{10}H_{16}$	0.303 ± 0.035a	0.161 ± 0.043b	–	0.189 ± 0.043b	–	–	0.049 ± 0.004c
	β-myrcene	$C_{10}H_{16}$	0.082 ± 0.009ab	0.095 ± 0.011a	0.069 ± 0.005bc	0.056 ± 0.004c	–	–	–
	α-thujene	$C_{10}H_{16}$	0.07 ± 0.009b	0.087 ± 0.008a	–	0.048 ± 0.004c	–	–	–
	Terpinolene	$C_{10}H_{16}$	0.076 ± 0.006b	0.09 ± 0.008a	–	0.05 ± 0.004c	–	–	–
	D-limonene	$C_{10}H_{16}$	0.084 ± 0.009a	0.095 ± 0.01a	–	0.077 ± 0.019a	–	0.088 ± 0.007a	0.049 ± 0.004b
	trans-*β*-ocimene	$C_{10}H_{16}$	–	–	0.563 ± 0.145a	–	–	–	–
	p-cymene	$C_{10}H_{14}$	–	–	–	0.047 ± 0.004a	–	–	0.049 ± 0.005a
	Sabinene	$C_{10}H_{16}$	0.082 ± 0.007a	0.093 ± 0.009a	–	0.052 ± 0.004b	–	–	0.049 ± 0.004b
	Eucalyptol	$C_{10}H_{18}O$	0.07 ± 0.009b	0.096 ± 0.009a	–	0.052 ± 0.003c	–	–	0.049 ± 0.004c
	β-ocimene	$C_{10}H_{16}$	0.069 ± 0.009b	0.086 ± 0.008b	3.806 ± 0.634a	0.049 ± 0.004b	–	0.088 ± 0.007b	0.049 ± 0.005b
	γ-terpinene	$C_{10}H_{16}$	0.089 ± 0.003a	0.093 ± 0.009a	–	0.053 ± 0.003b	–	–	–
	2-Carene	$C_{10}H_{16}$	0.072 ± 0.008b	0.088 ± 0.008a	0.062 ± 0.006bc	0.049 ± 0.003c	–	–	–
	Linalool	$C_{10}H_{18}O$	–	–	0.061 ± 0.006a	0.047 ± 0.004b	–	–	–
	1,3,8-p-menthatriene	$C_{10}H_{14}$	–	–	0.23 ± 0.06a	–	–	–	–
	(E,E)-2,6-dimethyl-2,4,6-octatriene	$C_{10}H_{16}$	–	–	0.064 ± 0.005a	–	–	–	–
	Camphor	$C_{10}H_{16}O$	0.069 ± 0.009ab	0.087 ± 0.008a	0.062 ± 0.005b	0.048 ± 0.004b	0.068 ± 0.009ab	0.088 ± 0.007a	0.053 ± 0.005b
	Santolina epoxide	$C_{10}H_{16}O$	–	–	–	–	–	–	0.049 ± 0.005a
Total monoterpene			2.07 ± 0.187b	2.093 ± 0.299b	5.041 ± 0.792a	1.128 ± 0.102bc	0.068 ± 0.009d	0.264 ± 0.022cd	0.442 ± 0.041cd
Sesquiterpene	(E)-*β*-farnesene	$C_{15}H_{24}$	–	–	0.062 ± 0.006b	–	–	0.088 ± 0.008a	–
	trans-*α*-bergamotene	$C_{15}H_{24}$	–	0.089 ± 0.009a	–	–	–	–	–
	Caryophyllene	$C_{15}H_{24}$	–	0.09 ± 0.009a	0.061 ± 0.006b	0.047 ± 0.004c	–	–	0.049 ± 0.005bc
	(Z,Z)-*α*-farnesene	$C_{15}H_{24}$	–	0.087 ± 0.008a	–	–	–	–	–
	Germacrene D	$C_{15}H_{24}$	–	0.09 ± 0.009a	–	–	–	–	–
	β-cadinene	$C_{15}H_{24}$	–	0.087 ± 0.008a	–	–	–	–	–
Total sesquiterpene			–	0.442 ± 0.044a	0.123 ± 0.011b	0.047 ± 0.004cd	0 ± 0	0.088 ± 0.008bc	0.049 ± 0.005cd

Aromatic compounds	Benzene	C_6H_6	0.035 ± 0.005bc	0.044 ± 0.004ab	0.031 ± 0.003c	0.024 ± 0.002c	0.034 ± 0.004bc	0.045 ± 0.004a	0.026 ± 0.002c
	Toluene	C_7H_8	0.035 ± 0.005ab	0.046 ± 0.005ab	0.032 ± 0.003ab	0.027 ± 0.004b	0.036 ± 0.005ab	0.059 ± 0.008a	0.059 ± 0.019a
	Ethylbenzene	C_8H_{10}	0.034 ± 0.005ab	0.044 ± 0.004a	0.031 ± 0.003b	0.024 ± 0.002b	0.035 ± 0.004ab	0.045 ± 0.004a	0.028 ± 0.003b
	p-xylene	C_8H_{10}	0.035 ± 0.005ab	0.045 ± 0.004a	0.031 ± 0.003b	0.025 ± 0.002b	0.036 ± 0.005ab	0.045 ± 0.004a	0.034 ± 0.006ab
	o-xylene	C_8H_{10}	0.034 ± 0.005ab	0.044 ± 0.004a	0.031 ± 0.003b	0.024 ± 0.002b	0.034 ± 0.004ab	0.044 ± 0.004a	0.027 ± 0.002b
	Styrene	C_8H_8	0.034 ± 0.005ab	0.044 ± 0.004a	–	–	0.034 ± 0.004ab	0.044 ± 0.004a	0.025 ± 0.002b
	1,2,3-trimethyl-benzene	C_9H_{12}	–	–	–	–	–	–	0.025 ± 0.002a
	Mesitylene	C_9H_{12}	–	–	–	–	–	–	0.024 ± 0.002a
	1-phenyl-1-pentanone	$C_{11}H_{14}O$	–	–	–	–	–	–	0.024 ± 0.002a
	α-methylstyrene	C_9H_{10}	0.035 ± 0.005a	–	–	0.024 ± 0.002b	0.034 ± 0.004a	–	–
	Benzaldehyde	C_7H_6O	–	0.044 ± 0.004a	0.031 ± 0.003b	–	–	–	0.025 ± 0.002c
	1-methyl-4-(1-methylethenyl)-benzene	$C_{10}H_{12}$	–	0.043 ± 0.004a	0.031 ± 0.003b	0.025 ± 0.002c	–	–	–
	α,α-dimethyl-benzenemethanol	$C_9H_{12}O$	0.035 ± 0.005b	0.044 ± 0.004a	–	–	–	–	–
	Naphthalene	$C_{10}H_8$	–	–	0.031 ± 0.003a	0.024 ± 0.002b	0.034 ± 0.004a	–	–
Total aromatic compounds			0.276 ± 0.036bc	0.395 ± 0.038a	0.247 ± 0.022bc	0.196 ± 0.016c	0.278 ± 0.035bc	0.283 ± 0.024bc	0.296 ± 0.032b
Green leaf volatiles	2-methyl-3-buten-2-ol	$C_5H_{10}O$	0.036 ± 0.005a	–	–	–	–	–	–
	3-octanol	$C_8H_{18}O$	–	–	–	–	–	–	0.024 ± 0.002a
	(Z)-3-hexen-1-ol	$C_6H_{12}O$	–	–	–	–	–	–	0.024 ± 0.002a
	(E)-2-octen-1-ol	$C_8H_{16}O$	–	–	–	–	–	0.058 ± 0.014a	–
	(E)-3-hexen-1-ol, acetate	$C_8H_{14}O_2$	0.058 ± 0.014a	–	0.033 ± 0.003b	–	–	0.058 ± 0.009a	0.028 ± 0.003b
	Acetic acid, hexyl ester	$C_8H_{16}O_2$	–	–	-	–	–	0.045 ± 0.004a	–
	(E)-2-hepten-1-ol	$C_7H_{14}O$	0.035 ± 0.005bc	0.043 ± 0.004ab	0.031 ± 0.003cd	0.024 ± 0.002d	–	0.044 ± 0.004a	0.024 ± 0.002d
	(E)-2-nonen-1-ol	$C_9H_{18}O$	–	–	–	–	–	–	0.025 ± 0.003a
	(E)-3-nonen-1-ol	$C_9H_{18}O$	0.035 ± 0.005a	0.044 ± 0.004a	0.036 ± 0.003a	0.024 ± 0.002b	–	0.044 ± 0.004a	–
	3-methyl-6-hepten-1-ol	$C_8H_{16}O$	–	–	0.031 ± 0.003a	–	–	–	–
	(Z)-3-nonen-1-ol	$C_9H_{18}O$	0.034 ± 0.005b	0.044 ± 0.004a	0.031 ± 0.003bc	0.024 ± 0.002c	–	0.044 ± 0.004a	0.024 ± 0.002c
	2-nonen-1-ol	$C_9H_{18}O$	–	–	–	–	–	–	0.024 ± 0.002a
Total green leaf volatiles			0.197 ± 0.031b	0.131 ± 0.013c	0.161 ± 0.014bc	0.071 ± 0.005d	0 ± 0	0.292 ± 0.027a	0.174 ± 0.016bc
Other compounds	2-butene	C_4H_8	–	–	–	0.024 ± 0.002a	–	–	0.025 ± 0.002a
	2-methyl-1-butanol	$C_5H_{12}O$	–	–	–	0.024 ± 0.002b	–	0.044 ± 0.004a	0.025 ± 0.002b
	Ethanol	C_2H_6O	–	–	0.031 ± 0.003b	0.024 ± 0.002c	–	0.044 ± 0.004a	–
	Acetone	C_3H_6O	0.035 ± 0.005abc	0.046 ± 0.004ab	0.032 ± 0.003bc	0.025 ± 0.002c	0.038 ± 0.004abc	0.048 ± 0.005a	0.04 ± 0.007ab
	Acetic acid, methyl ester	$C_3H_6O_2$	–	–	–	–	–	–	0.025 ± 0.002a

3-pentanol	$C_5H_{12}O$	–	0.043 ± 0.004a	–	0.024 ± 0.002c	0.034 ± 0.004b	–	–
2-methyl-furan	C_5H_6O	–	0.044 ± 0.004a	–	–	–	0.044 ± 0.004a	0.025 ± 0.002b
(S)-1,3-butanediol	$C_4H_{10}O_2$	0.034 ± 0.005b	–	0.031 ± 0.003bc	–	–	0.045 ± 0.004a	0.025 ± 0.002c
Ethyl acetate	$C_4H_8O_2$	–	–	–	–	–	–	0.025 ± 0.003a
2-Butanol	$C_4H_{10}O$	–	–	–	–	–	–	0.025 ± 0.002a
3-methyl-hexane	C_7H_{16}	0.035 ± 0.004ab	0.044 ± 0.005a	–	0.024 ± 0.002c	0.035 ± 0.004ab	0.044 ± 0.004a	0.025 ± 0.002bc
Bi-2-cyclohexen-1-yl	$C_{12}H_{18}$	–	–	–	–	–	–	0.025 ± 0.002a
2,3,4-trimethyl-hexane	C_9H_{20}	–	–	–	–	0.034 ± 0.004a	–	–
2,2,3,4-tetramethyl-pentane	C_9H_{20}	–	–	–	–	0.035 ± 0.004a	–	0.025 ± 0.003b
2,7-dimethyl-1-octanol	$C_{10}H_{22}O$	–	0.044 ± 0.004a	0.031 ± 0.003b	–	0.034 ± 0.004ab	0.044 ± 0.004a	0.031 ± 0.007b
2-propyl-1-heptanol	$C_{10}H_{22}O$	–	0.043 ± 0.004a	0.031 ± 0.003bc	0.024 ± 0.002c	–	–	0.024 ± 0.002c
2-butyl-1-octanol	$C_{12}H_{26}O$	0.035 ± 0.005bc	0.046 ± 0.005a	0.031 ± 0.003c	0.024 ± 0.002c	–	0.045 ± 0.004ab	0.024 ± 0.002c
4-methyl-1-undecene	$C_{12}H_{24}$	–	–	0.031 ± 0.003a	–	–	–	–
Total other compounds		0.138 ± 0.018b	0.31 ± 0.03a	0.217 ± 0.019b	0.192 ± 0.015b	0.313 ± 0.039a	0.357 ± 0.031a	0.369 ± 0.038a
Total BVOCs		2.683 ± 0.257cd	3.373 ± 0.386cd	5.791 ± 0.8b	1.635 ± 0.115d	2.039 ± 0.337d	7.769 ± 0.989a	4.391 ± 0.922bc

Values are represented as the mean ± standard error (SE) (n = 6). Different lowercase letters within a row indicate significant differences among different plant species bases on Tukey's test ($p < 0.05$). "–" indicates was not detected.
